# Regulation of efferocytosis as a novel cancer therapy

**DOI:** 10.1186/s12964-020-00542-9

**Published:** 2020-05-05

**Authors:** Yunxiang Zhou, Yihan Yao, Yongchuan Deng, Anwen Shao

**Affiliations:** 1 Department of Surgical Oncology, The Second Affiliated Hospital, School of Medicine, Zhejiang University, Hangzhou, China; 2 Department of Neurosurgery, The Second Affiliated Hospital, School of Medicine, Zhejiang University, Hangzhou, China

**Keywords:** Efferocytosis, Tumor progression, Antitumor therapy, Immunosuppression, Phosphatidylserine, Phosphatidylserine receptor, CD47

## Abstract

Efferocytosis is a physiologic phagocytic clearance of apoptotic cells, which modulates inflammatory responses and the immune environment and subsequently facilitates immune escape of cancer cells, thus promoting tumor development and progression. Efferocytosis is an equilibrium formed by perfect coordination among “find-me”, “eat-me” and “don’t-eat-me” signals. These signaling pathways not only affect the proliferation, invasion, metastasis, and angiogenesis of tumor cells but also regulate adaptive responses and drug resistance to antitumor therapies. Therefore, efferocytosis-related molecules and pathways are potential targets for antitumor therapy. Besides, supplementing conventional chemotherapy, radiotherapy and other immunotherapies with efferocytosis-targeted therapy could enhance the therapeutic efficacy, reduce off-target toxicity, and promote patient outcome.

**Video abstract**

**Video abstract**

## Background

Apoptosis is a mechanism of programmed cell death by which tissues self-renew and maintain homeostasis [1]. During apoptosis, phagocytes rapidly recognize and engulf dying cells before intracellular components are released. Subsequently, the membrane integrity of apoptotic cells is maintained, hence avoiding the exposure of immunogenic materials and the subsequent inflammatory responses [2]. This physiologic process, referred to as efferocytosis, efficiently removes apoptotic cells without subsequent secondary necrosis and damages [3]. Efferocytosis is induced by several physiological or pathological conditions and plays a crucial role in tissue differentiation, repair, and the resolution of inflammation [4]. Because of the pleiotropic role of efferocytosis, disruption and dysregulation of this process are associated with many pathological states, which may trigger some diseases. Previous research has linked efferocytosis to inflammatory diseases [5], autoimmune diseases [6] and atherosclerosis [7, 8].

Apoptotic cell removal is accomplished by either professional or non-professional phagocytes [9]. Professional phagocytes include macrophages and immature dendritic cells, which are recruited following the onset of apoptosis with a relatively faster and better motile ability in engulfment; while non-professional phagocytes are tissue-resident cells neighboring dying cells (e.g., epithelial cells, endothelial cells, fibroblasts and some stromal cells) and are comparatively slower in ingesting dead cells [10–12].

Tumor-associated macrophages are M2-polarized macrophages and a type of phagocyte involved in efferocytosis [13, 14]. Recent studies have demonstrated the vital role of efferocytosis in the tumor microenvironment, progression, and metastasis of tumors [15, 16]. Following engulfment, tumor-associated macrophages increase the production of anti-inflammatory cytokines and Treg cells (regulatory T cells), while inhibiting the production of pro-inflammatory cytokines and effector T cells [17–19]. Therefore, the immunologically silent clearance of apoptotic cells promotes inflammation resolution and immune suppression, which provide cancer cells with an environment to escape from immunological surveillance hence promoting tumor progression [1, 20]. This review explores the function and underlying mechanisms of efferocytosis in tumor progression and summarizes the promising targets and novel strategies for cancer therapy.

## Biological mechanisms of efferocytosis

### Recognition

Efferocytosis includes complicated and coordinated molecular communication and involves several signaling pathways that culminate in phagocytosis and clearance of dying cells. The first step in efferocytosis is recognition of the targeted apoptotic cells by phagocytes through “find-me” signals such as lipids, proteins, peptides, and complex structures released by dying cells [21]. These signals act as chemo-attractants for scavenger cells recruited to the apoptotic site during the early stages of efferocytosis. The first chemotactic factor to be identified as a “find-me” signal was a covalent dimer of the ribosomal protein S19 [22]. Lysophosphatidylcholine is a lipid attraction signal produced by apoptotic cells through caspase-3 mediated activation of the calcium-independent phospholipase A2 and stimulates the migration of monocytes and primary macrophages [23, 24]. Also, sphingosine-1-phosphate (S1P) is another lipid chemotactic factor that acts as a “find-me” signal in efferocytosis. S1P triggers the chemotaxis of macrophages to engulf apoptotic cells [25]. As a member of the chemokine family, CX3CL1 is also a “find-me” signal released by lymphocytes after apoptosis and attracts macrophages to the apoptotic site [26]. Elliott et al. demonstrated the role of extracellular nucleotides in phagocytosis and apoptotic cell recognition, as the release of ATP and UTP from apoptotic cells elicit an attraction signal through their receptor P2Y_2_ on phagocytes [27].

### Engulfment

Following recognition and migration of phagocytes, apoptotic cells are engulfed by phagocytes. Engulfment is mediated by a series of molecular events called “eat-me” signals such as phosphatidylserine (PS) and calreticulin. These signals are unique surface markers on apoptotic cells identified by arrived phagocytes and enable them to exert a subsequent phagocytic function. The most common and widely studied “eat-me” signal is PS, which is usually confined to the inner leaflet of the plasma membrane but migrates to the outer leaflet during apoptosis [28, 29]. Apoptotic cells typically have exposed PS, phagocytes recognize and combine with this signal before the onset of engulfment.

Previous studies have reported that phagocytes can directly bind to PS on apoptotic cells through numerous PS receptors. These receptors include T-cell immunoglobulin mucin (TIM) family (TIM-1, TIM-3, and TIM-4) [30, 31], brain-specific angiogenesis inhibitor 1 (BAI-1) [32], stabilin-2 family members [33], CD300 family members (CD300b and CD300f) [34, 35] and receptor for advanced glycation end products (RAGE) [36]. Alternatively, PS can indirectly bind to receptors on phagocytes via bridging molecules. For example, TAM receptors are a type of indirect PS receptors, and growth arrest-specific 6 (Gas6) and Protein S are bridging molecules that facilitate the binding of PS on apoptotic cells to TAM receptors (Tyro3, Axl and MerTK) on phagocytes [37]. Further, Gas 6 binds to all three receptors, while Protein S only binds to Tyro3 and MerTK [38]. Milk fat globule epidermal growth factor-8 (MFG-E8) bridges between PS and α_v_β_3_/α_v_β_5_ integrins, another type of indirect PS receptors [39]. Besides, the SCARF1 scavenger receptor on phagocytes uses C1q as a complement component to recognize PS on apoptotic cells [40]. Inversely, phagocytes can identify live and normal cells by detecting “don’t-eat-me” signals, which protects the cells from being engulfed. The most widely studied ligands of “don’t-eat-me” signals are CD47 [4] and CD31 [41].

Following the “eat-me” signals recognition and tethering, cytoskeletal rearrangement occurs within phagocytes, leading to cell motility and phagosome formation to complete the process of engulfment. This process is mediated by ELMO/Dock180/Rac1 pathway [40, 41], which is proved to be downstream of PS receptors such as BAI-1 [30]. In addition, TAM receptors and α_v_β_5_ integrin have been confirmed to have an effect on Rac1 activation and eventually causing phagocytosis [42]. However, mechanisms of some other receptors in post-receptor activation signaling are still unclear.

### Immunomodulation

The clearance of apoptotic cells occurs via their interaction with phagocytes, which contributes to tissue homeostasis. This process is accompanied by the secretion of a series of anti-inflammatory cytokines, including transforming growth factor-beta (TGFβ), interleukin (IL)-10, prostaglandin E2 (PGE2) and platelet-activating factor (PAF). Meanwhile, the production of the pro-inflammatory cytokines IL-1β, tumor necrosis factor-alpha (TNF-α), and IL-12 is inhibited [42, 43]. This mechanism occurs via the reduction of M1 macrophage induced production of pro-inflammatory cytokines and suppression of the nuclear factor-kappa B (NF-ĸB) signaling [44]. In contrast, the anti-inflammatory response of M2 macrophages is enhanced [45]. Besides, TAM receptor activation dampens pro-inflammatory Toll-like receptor (TLR) signaling by upregulating the expression of suppressor of cytokine signaling (SOCS) 1 and SOCS3. Furthermore, the enhancement of efferocytosis is always in line with the decreased activation and function of CD8^+^ and CD4^+^ effector T cells as well as increased Treg cells-mediated immunosuppression [17, 46–49]. Indeed, CD8^+^ and CD4^+^ T cells play a critical role in tumor rejection response [50]. Particularly, CD8^+^ cytotoxic T cells are acknowledged to recognize tumor-specific antigens and target cancer cells, leading to the shrinkage of tumor [51].

**Fig. 1:**
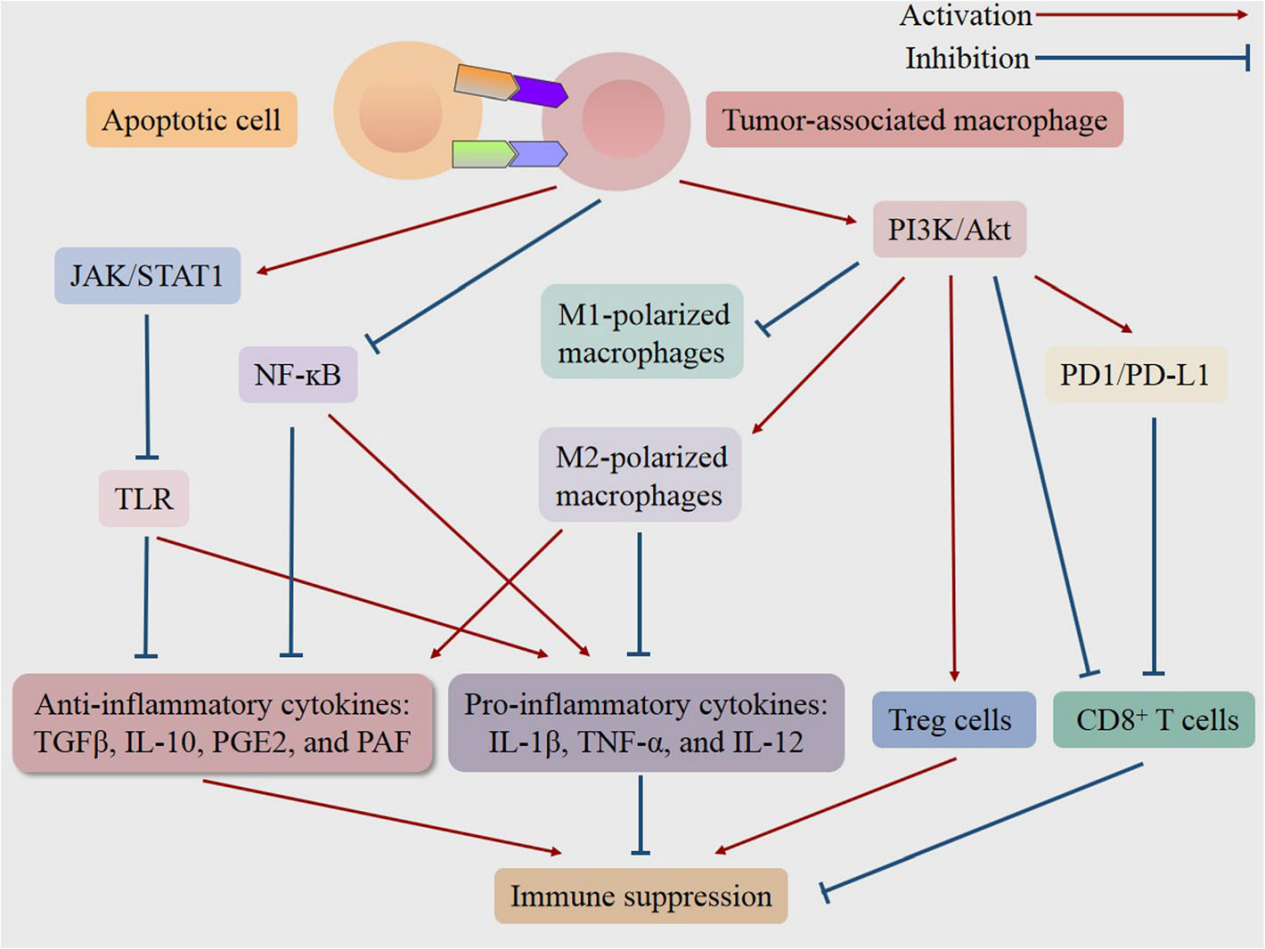
Schematic representation of signaling pathways in the efferocytosis-induced immune suppression for tumor progression. The engulfment of apoptotic cells by tumor-associated macrophages triggers a series of signaling pathways, subsequently induces M2 polarization of macrophages while inhibiting M1 polarization, increases Treg cells while decreasing CD8+ T cells, and thereby resulting in the inflammation resolution and immune suppression, which may provide an environment for cancer to escape from immunological surveillance and promote tumor progression. NFκB = factor-κ-gene binding; JAK/STAT1 = Janus kinase/signal transducers and activators of transcription 1; PI3K/Akt = phosphatidylinositol 3 kinase/protein-serine-threonine kinase; PD-1/PD-L1 = programmed death-ligand 1/programmed cell death protein 1; TLR = Toll-like receptor; IL = interleukin; TGFβ = transforming growth factor-beta; PGE2 = prostaglandin E2; PAF = platelet-activating factor; TNF-α = tumor necrosis factor-alpha; Treg cells = regulatory t cell

Taken together, efferocytosis hinders the inflammatory response and modulates the immune environment, which facilitates immune escape and promotes tumorigenesis and progression **(**Fig. 1**)** [52]. Conversely, inhibition of efferocytosis in the context of cancer results in the release of intracellular components of dying cells. And the succeeding exposure of immunogenic materials triggers the robust innate and adaptive immune responses against cancer, due to the secretion of pro-inflammatory cytokines, the accumulation of inflammatory cells, as well as the enhanced recognition and presentation of tumor-specific antigens [1, 2, 20, 51].

## The role of efferocytosis in the tumor microenvironment

The mechanisms of several efferocytosis-associated molecules and signaling pathways have been elucidated in the past few decades.

### TAM receptors

TAM receptors promote the tumor microenvironment by promoting remodeling of the extracellular matrix and the release of factors conducive to cell proliferation, migration, and angiogenesis [53]. Under normal physiological conditions, TAM receptors are principally expressed on natural killer (NK) cells and antigen-presenting cells (APCs) such as macrophages and immature dendritic cells [54]. However, TAM receptors are overexpressed in various cancers, including lung cancer, leukemia, gastrointestinal cancer, and breast cancer, and the overexpression results in enhanced efferocytosis and a worse cancer outcome [55–57]. The underlying mechanisms of TAM receptors, particularly Axl and MerTK, have been widely researched.

The oncogenic and cancer-promoting mechanisms of MerTK are associated with the classic cell proliferation pathways phosphatidylinositol 3 kinase (PI3K)/protein-serine-threonine kinase (Akt) and mitogen-activated protein kinase (MAPK)/extracellular signal-regulated kinase (ERK) [55]. Through the PI3K/Akt signaling axis, MerTK induces polarization of M2 macrophages while inhibiting polarization of M1 macrophages, and thereby resulting in the resolution of inflammation [58]. M2 macrophages play a crucial role in the cancer microenvironment by facilitating tumor progression and invasion due to their immunosuppressive characteristics [59]. In addition to innate immunity, MerTK is involved in the modulation in adaptive immunity as genetic ablation of MerTK parallels increased intratumoral CD8^+^ T lymphocytes and promoted lymphocyte proliferation [48]. Moreover, MerTK-driven efferocytosis also induces the expression of the programmed death-ligand 1 (PD-L1), a molecule that promotes tumor escape, and therefore enhances the immune suppression microenvironment for cancer cells [57]. Notably, MerTK is essential for TIM-4 induced efferocytosis [60]. Since TIM-4 lacks an extensive intracellular domain, it works in tandem with MerTK to facilitate the phagocytosis of apoptotic cells [60]. Furthermore, it has been shown in many malignant tumors that MerTK is involved in the resistance of multiple anticancer therapies. In non-small cell lung cancer, for instance, MerTK participates in drug-resistance of epidermal growth factor receptor (EGFR) inhibitors [61].

Axl is typically activated by the Gas6 ligand, and the affinity of Gas6 for Axl is higher than to Tyro3 and MerTK receptors [62]. The Gas6/Axl signaling pathway influences cancer development and progression through its effect on tumor cell proliferation, invasion, metastasis, epithelial-mesenchymal transition (EMT), and angiogenesis [38, 63, 64]. A previous study demonstrated that Axl is required for EMT, which promotes metastasis of HER-2 positive breast cancer [65]. Several signaling pathways, including Janus kinase (JAK)/signal transducers and activators of transcription 1 (STAT1), PI3K/Akt, and NF-ĸB, MAPK/ERK are downstream of the Gas6/Axl pathway [66]. Therefore, macrophage polarization induced by the PI3K/Akt signaling axis is also involved in the cancer-promoting mechanisms of Axl. Moreover, Axl could mediate resistance to chemotherapy [67], radiotherapy, and targeted molecular therapy in many cancers [68]. For instance, overexpression of Axl promotes resistance to EGFR-tyrosine kinase inhibitor (TKI) through the PI3K/Akt and MAPK/ERK signaling pathways [69]. In small-cell lung cancer, Axl promotes primary and acquired resistance to WEE1 (WEE1 G2 checkpoint kinase) inhibition. This resistance occurs via activation of another G2-checkpoint protein, the checkpoint kinase 1 (CHK1), which is a parallel pathway for the repair of DNA damage [70].

Furthermore, TAM receptors are characterized as ubiquitylation substrates for the E3 ligase casitas B-lineage lymphoma-b (Cbl-b), which suppresses antitumor activities of NK cells, CD8^+^ T cells and CD4^+^ T cells while enhances Treg immunosuppressive activity [46, 47, 71, 72]. Consequently, the inhibition of Cbl-b by blocking TAM receptors has the potential to boost immunity against cancer.

### Direct PS receptors

Among the TIM family, TIM-4 is expressed on the surface of phagocytes and is tightly involved in the efferocytosis [73, 74]. Although the overall function of TIM-4 remains obscure, several pieces of evidence suggest that TIM-4 is involved in tumor progression. Overexpression of TIM-4 has been shown to promote the proliferation of non-small cell lung carcinoma [75]. Also, a previous study demonstrated that TIM-4 attenuates the effect of chemotherapy and increases immune tolerance to cancer via interaction with AMPKα1 [76]. A recent study explored the role of TIM-4 in colorectal cancer and reported that TIM-4 promotes angiogenesis by upregulating vascular endothelial growth factor (VEGF). TIM-4 also recruits tumor-associated macrophages through the PI3K/Akt signaling pathway, thereby promoting cancer progression [77]. The roles of other direct PS receptors (BAI-1, stabilin-2 family members, and CD300 family members) remain elusive.

### Bridging molecules

Gas6 bridges between PS and TAM receptors, thereby promoting cancer cell proliferation and migration [78]. Notably, leukocytes infiltrating through cancerous tissues upregulate Gas6, which contributes to tumor growth and invasion [79]. The role of the Gas6/Axl signaling pathway in cancers has been described in the “TAM receptors” section.

MFG-E8 bridges between PS and α_v_β_3_/α_v_β_5_ integrins [39] and its expression is upregulated in tumors [80]. MFG-E8 attenuates inflammation and increases Treg cells, efferocytosis, angiogenesis, allograft tolerance, tumorigenicity, and cancer metastasis [81, 82]. Moreover, overexpression of MFG-E8 is negatively associated with prognosis in various cancers including breast, colorectal and esophageal cancers [83–85]. According to Yamada et al.*,* MFG-E8 promotes angiogenesis by upregulating the expression of VEGF and endothelin (ET)-1 in bone marrow-derived mesenchymal stromal cells to trigger tumor progression in melanomas [86]. Besides, MFG-E8 enhances M2 polarization of macrophages [86, 87], and blockade of MFG-E8 enhances antitumor effector T cells but inhibits Treg cells, leading to tumor destruction [17].

## The implication of efferocytosis in cancer therapy

Given the vital role of efferocytosis in the tumor microenvironment, progression, and metastasis, efferocytosis-targeted approaches could offer a novel therapeutic strategy in tumorigenesis and cancer management [1, 20]. We have summarized some representative agents of efferocytosis-targeted therapy in Table. 1. Also, chemotherapy and radiotherapy induce apoptosis of cancer cells and increase the subsequent efferocytosis, which suppresses inflammatory responses. Therefore, combining these traditional therapies with efferocytosis-targeted therapy or other types of immunotherapy could enhance their efficacy and improve patient outcomes [73].

**Table 1:** Representative agents of efferocytosis-targeted therapy

Agents	Sub-types	Mechanisms or effects	References
Annexin A5	Natural occurring ligands for PS	Inhibit PS-dependent phagocytic activity, produce proinflammatory mediators and not produce sufficient factors related with tissue repair.	[20]
Bavituximab	Antibody binding specifically to PS		[88–90]
UNC2025	Tyrosine kinase inhibitor against MerTK	Cause visual impairment, produce proinflammatory mediators and not produce sufficient factors related with tissue repair.	[91]
BGB324, SGI-7079, TP-0903, DAXL-88, DP3975 and NA80xl	small-molecule TKIs against Axl	Produce proinflammatory mediators and not produce sufficient factors related with tissue repair; some TKIs cause fatigue, diarrhea, hypertension, hematologic events, and palmar-plantar erythrodysesthesia syndrome.	[38, 92]
GL21.T	Nucleotide aptamer binding specifically to Axl	Produce proinflammatory mediators and not produce sufficient factors related with tissue repair.	[38]
YW327.6S2, D9 and E8	Monoclonal antibody binding specifically to Axl		[38]
Soluble Axl	Inhibiting the transmembrane Axl and Gas6 signaling		[38, 93]
Celastrol, dihydroartemisinin	Natural compound inhibiting Axl		[38, 94, 95]
Warfarin	Oral anticoagulant suppressing Gas6 activity	Cause hemorrhage, produce proinflammatory mediators and not produce sufficient factors related with tissue repair.	[47]
Small interfering RNA	Nucleotide aptamer binding specifically to MFG-E8	Produce proinflammatory mediators and not produce sufficient factors related with tissue repair.	[96]
HMGB1, extracellular matrix ligands	Inhibiting α_v_β_3_/α_v_β_5_ integrins		[97, 98]
B6H12.2, BRIC126	Anti-CD47 antibodies	Induce the phagocytosis of live and normal cells.	[49, 99, 100]
ICAM-1	Transmembrane glycoprotein inhibiting efferocytosis	Not mentioned.	[101]

*Abbreviations*: PS, phosphatidylserine; TKI, tyrosine kinase inhibitor; MFG-E8, Milk fat globule epidermal growth factor-8; CD, cluster of differentiation; Gas, growth arrest-specific protein 6; ICAM-1, intercellular cell adhesion molecule-1; HMGB1, high-mobility group box 1

### Blockade of “eat-me” signaling

Notably, “find-me” signals are not tumor-specific. More research has, therefore, focused on therapies targeted to the “eat-me” signaling pathway, among which the previously described PS signaling is the most common and the most widely studied.

#### PS targeting

Several PS targeting agents, such as annexin proteins and PS targeting antibodies, have been widely studied [1]. Annexin proteins, the naturally occurring ligands for PS, saturate and block the externalized PS, thus inhibiting the “eat-me” signaling pathway [103]. This blockage triggers a pro-inflammatory response, increases the immunogenicity of apoptotic tumor cells, and shifts the immunosuppressive environment towards an antitumor response [20, 88, 89]. PS targeting antibodies specifically bind to PS with high affinity. As PS is also expressed in vascular endothelial cells, these antibodies not only target PS-expressing tumors but also target tumor blood vessels [90, 104, 105]. The interaction between PS targeting antibodies and exposed PS increases the expression of inflammatory cytokines and reduces the expression of immunosuppressive myeloid-derived suppressor cells [106]. Besides, PS targeting antibodies induce the polarization of M1 macrophages and recruitment of mature dendritic cells, leading to an increase of tumor-specific cytotoxic T cells [106]. When used in combination with either chemotherapy, radiotherapy, or immune checkpoint antibodies (anti-CTLA-4 and anti-PD-1), PS targeting agents have been shown to facilitate the curative effect of these therapies [20, 88]. As such, pre-clinical agents associated with PS targeting antibodies such as Annexin A5 of annexin proteins and 3G4, 2aG4 and chimeric 1 N11 have been developed [20]. Multiple clinical trials of bavituximab, a PS targeting antibody, have also been carried out [107–109].

However, subsequent phase II study and phase III trial did not provide evidence on the substantial improvement of efficacy following the addition of bavituximab compared to the chemotherapy alone group [54, 110]. Besides efferocytosis, PS targeting therapy also interferes with the function of antigen-presenting cell (APCs) and induces non-selective inhibition of all PS-dependent phagocytic activity. Thus, PS inhibition may cause other harmful side effects on the body [54]. Notably, PS receptor-blocking approaches also inhibit PS signaling pathway.

#### TAM targeting

TAM receptors play a pleiotropic role in tumor pathophysiology and drug resistance. Previous studies have reported that all three TAM receptors are overexpressed in various cancers. This overexpression promotes oncogenic signaling and efferocytosis, resulting in a worse cancer outcome [55–57].

The Axl inhibitors potentiate the apoptosis of live cancer cells, reduce migration and invasion of tumor cells, and suppress efferocytosis [92]. Previous studies have also reported that Axl targeting synergizes with chemotherapy and other targeted therapies such as VEGF, EGFR, PI3K, PARP (poly ADP-ribose polymerase), and HER2 inhibitors to promote therapeutic efficacy [111–113]. There are currently five types of Axl targeting agents under development. These agents include small-molecule TKIs, nucleotide aptamers, monoclonal antibodies (mAbs), soluble receptors, and several natural compounds [38]. Research on reputable Axl inhibitors, especially the TKIs, has now progressed into clinical trial phases [38]. Nonetheless, Axl TKIs have increased clinical off-target toxicity and drug resistance [93, 94]. Nucleotide aptamers and mAbs are emerging therapy with higher affinity and specificity, and lower toxicity and drug resistance [91, 95, 114, 115], although their application is currently at preclinical stages [38]. Other than the transmembrane form, Axl can also exist in a soluble form once it is cleaved in the extracellular domain. Soluble Axl binds to Gas6 or Axl itself, thereby inhibiting the transmembrane Axl and Gas6 signaling pathway [116]. Besides, it has been shown that natural compounds such as celastrol [117] and dihydroartemisinin [118] show therapeutic potential through Axl inhibition.

MerTK can induce intrinsic and adaptive resistance of Axl-targeted agents, which advocates a dual targeting of Axl and MerTK for the hindrance of downstream signaling and tumor growth [119]. However, the ablation of MerTK may cause visual impairment [96, 120]. Hence, the safety of MerTK-targeted therapies should be explored further. Several preclinical studies have reported similar adaptive responses caused by single TKI therapy, and co-targeting of the receptor tyrosine kinase family could, therefore, be a novel strategy for overcoming drug resistance and increasing efficacy [61, 121–125]. Notably, cells of the innate immune system are involved in the initiation and regulation of adaptive immune response. Co-targeting innate immune checkpoints such as TAM receptors may thus enhance the recruitment and activation of adaptive immune cells and increase the therapeutic efficacy compared to the single adaptive immune checkpoint-targeted therapy [54].

#### MFG-E8 targeting

Although the administration of MFG-E8 nucleotide aptamer alone may not be effective enough for averting tumor progression and boosting immunity [97]. Previous studies have demonstrated that the down-regulation of MFG-E8 increases the sensitivity of tumor cells to TKIs and cytotoxic agents in vitro [98, 126]. Besides, the combination of chemotherapy and MFG-E8 RNA interference contributes to sustained inhibition of tumor survival and growth [97]. These synergistic actions could be attributed to several mechanisms since the down-regulation of MFG-E8 signaling results in several different effects, including (1) decrease in chemotherapy resistance of tumor cells; (2) inhibition of MFG-E8-mediated efferocytosis; (3) blockage of VEGF-induced angiogenesis; (4) enhanced cross-presentation between the dying tumor cells and dendritic cells; (5) reduced Treg cells and increased activation and function of CD4^+^ and CD8^+^ effector T cells [17, 97, 127, 128]. Indeed, MFG-E8 bridges between PS exposed on apoptotic cells and α_v_β_3_/α_v_β_5_ integrins expressed on macrophages [39, 129]. Therefore, integrin-targeted molecules such as extracellular matrix ligands, high-mobility group box 1, and inhibitory antibodies also suppress efferocytosis [99, 130].

In addition to the blockade of the above “eat-me” signaling pathways, targeting the TIM receptor family and Gas6, which are the direct PS receptors and bridging molecule for efferocytosis respectively, could also improve the current immunotherapies [1, 47]. However, the blockade of “eat-me” signaling pathways may produce excessive pro-inflammatory mediators and fail to produce sufficient factors for tissue repair [131, 132], which compromises on the rationality of this therapeutic strategy.

### Blockade of “don’t-eat-me” signaling

Efferocytosis is an equilibrium formed following proper coordination among “find-me”, “eat-me” and “don’t-eat-me” signals [73]. The “don’t-eat-me” signaling are primarily emitted by CD47, whose receptor is signal regulatory protein-α (SIRP-α), a protein expressed on the surface of phagocytes [73, 133]. Previous studies have revealed that CD47 is overexpressed (approximately three-fold compared to healthy cells) on the plasma membrane of all human cancers and enables cancer cells to evade phagocytosis [49, 134]. Notably, the expression of CD47 mRNA is negatively correlated with patient survival rates [134]. Dysregulation of CD47, therefore, affects tumor-associated efferocytosis and represents a promising therapeutic strategy.

Anti-CD47 mAbs facilitate phagocytosis of cancer cells [134]. In addition, anti-CD47 mAbs decrease the ability of Treg cells to overcome immune evasion by cancer cells and increase the capacity of CD8^+^ T cells to exhibit an effective antitumor cytotoxic function [49]. Furthermore, anti-CD47 mAbs prevent or eliminate metastatic lesions and circulating tumor cells [135, 136]. Consequently, the blockade of CD47 signals inhibits the growth and metastasis of human tumors [134]. Since CD47 is also expressed on normal cells at varying levels, looming safety concerns on targeting CD47 should be addressed [100]. Previous studies have revealed that anti-CD47 mAbs could produce potent antitumor responses without causing any severe side effects, even at doses that exceed the minimum effective dose. This observation could be attributed to the lack of secondary prophagocytic “eat-me” signals on the surface of healthy cells [101, 134]. Under cellular stress, however, the calreticulin of healthy cells may translocate to the cell surface and result in phagocytosis of healthy cells by nearby macrophages. Thus, targeting CD47 should be applied with caution in the context of recent ongoing inflammatory or cytotoxic treatments [137, 138]. The findings of a study by Willingham et al. point out that a better efficacy of anti-CD47 mAbs therapy is correlated with smaller tumor size at the onset of treatment. The study further proposes that the optimal time to effect an anti-CD47 therapy is after maximal cytoreductive surgery [134].

Combination of anti-CD47 mAbs and other antitumor antibodies such as trastuzumab (anti-HER2 antibody), rituximab (anti-CD20 antibody), alemtuzumab (anti-CD52 antibody), and cetuximab (anti-epidermal growth factor receptor antibody) elicits a synergistic effect. This effect may be attributed to a magnifying effect of Fc receptor-dependent phagocytosis by the second antitumor antibody [49, 102, 134]. Such a combined therapy does not only result in increased therapeutic efficacy but also possesses several advantages. These advantages include (1) decreased off-target toxicity compared to chemotherapy; (2) reduced potential antibody toxicity compared to monotherapies; (3) enhanced cancer cell elimination, even when the epitope of a single drug target mutates [49]. Several anti-CD47 mAbs have so far been developed, including B6H12.2 and BRIC126 [49, 139, 140], and efficacy studies are ongoing. However, the research is based on xenotransplantation models, and more experimental studies and clinical trials should, therefore, be done to validate the reported efficacies.

### Intercellular cell adhesion molecule-1

Intercellular cell adhesion molecule-1 (ICAM-1) is a transmembrane glycoprotein of the immunoglobulin supergene family, which is expressed in all leukocytes and is a ligand for β2 integrins [141, 142]. ICAM-1 is also expressed on the cell surface of many cancer types and facilitates tumor progression and metastasis [143]. ICAM-1 inhibits the efferocytosis of apoptotic tumor cells through the suppression of the PI3K/Akt signaling pathway, and the downregulation of efferocytosis leads to the decrease of M2 macrophage polarization, consequently inhibiting tumor progression and tumor metastasis [143, 144]. Therefore, the role of ICAM-1 in efferocytosis makes it a promising target for cancer treatment. Other agents that alter the polarization of tumor-associated macrophages are also of therapeutic potential.

### Combined inhibition of apoptosis and secondary necrosis

A recent study [52] described two distinct mechanisms for cell death: apoptosis and secondary necrosis, both of which affect the tumor microenvironment in different ways. In the study, inhibition of efferocytosis did not suppress the production of tumor myeloid-derived suppressor cells, Treg cells, and some immunosuppressive mediators. Further research uncovered that this phenomenon could be because decreased efferocytosis induced secondary necrosis of apoptotic cells, which stimulated the expression of inflammation-resolving factor indoleamine-2,3-dioxygenase (IDO) 1, leading to the restoration of the immunosuppressive environment for cancer cells. The results indicate that apoptotic and necrotic cancer cells promote tumor progression through efferocytosis and IDO1 respectively. Combined inhibition of these two processes showed a better result in tumor regression and inhibition of metastasis [52].

## Conclusion and future perspectives

Efferocytosis inhibits inflammatory responses, modulates the immune environment, and facilitates immune escape of cancer cells. Subsequently, efferocytosis promotes tumor development and progression. Efferocytosis is an equilibrium formed by perfect coordination among “find-me”, “eat-me” and “don’t-eat-me” signals. Among the three signals, the “find-me” signals do not exhibit specificity for antitumor targeting, and many studies have, therefore, focused on the “eat-me” and “don’t-eat-me” signals. These signaling pathways influence cancer development and progression by affecting the proliferation, invasion, and metastasis of tumor cells, epithelial-mesenchymal transition, and angiogenesis. Thus, the signals represent potential therapeutic targets for cancer treatment.

On the other hand, chemotherapy and radiotherapy induce apoptosis of cancer cells and increases the subsequent efferocytosis. Combining these traditional therapies with efferocytosis-targeted therapy could, therefore, enhance the efficacy and promote patient outcomes. Single TKI therapy also elicits adaptive responses and drug resistance, and co-targeting receptors of the tyrosine kinase family are promising for overcoming TKI-related drug resistance and increasing treatment efficacy. Also, the combined therapies could result in reduced side effects. However, the mechanisms underlying efferocytosis are obscure; for instance, the Tyro3 pathway has not been extensively explored. Besides, no compelling evidence on the efficacy of several efferocytosis inhibitors such as bavituximab has been established, and the potential clinical off-target toxicity may limit the clinical application. Furthermore, the safety of some efferocytosis-associated including MerTK inhibitors and anti-CD47 mAbs needs to be further studied.

## Data Availability

Not applicable.

## Abbreviations

S1P: Sphingosine-1-phosphate
PS: Phosphatidylserine
TIM: T-cell immunoglobulin mucin
BAI-1: Brain-specific angiogenesis inhibitor 1
TAM: Tyro3, Axl and MerTK
Gas: Growth arrest-specific protein 6
TGFβ: Transforming growth factor-beta
PGE2: Prostaglandin E2
PAF: Platelet-activating factor
IL: Interleukin
NFκB: Factor-κ-gene binding
TNF-α: Tumor necrosis factor-alpha
TLR: Toll-like receptor
SOCS: Suppressor of cytokine signaling
APCs: Antigen-presenting cells
JAK/STAT1: Janus kinase/signal transducers and activators of transcription 1
PI3K/Akt: Phosphatidylinositol 3 kinase/protein-serine-threonine kinase
PD-1: Programmed death-ligand 1
PD-L1: Programmed cell death protein 1
Treg cells: Regulatory T cell
TKI: Tyrosine kinase inhibitor
MFG-E8: Milk fat globule epidermal growth factor-8
CD: Cluster of differentiation
ICAM-1: Intercellular cell adhesion molecule-1
EGFR: Epidermal growth factor receptor
EMT: Epithelial-mesenchymal transition
CHK1: Checkpoint kinase 1
NK cell: Natural killer cell
VEGF: Vascular endothelial growth factor
ET: Endothelin
CTLA-4: Cytotoxic T-lymphocyte-associated protein 4
PARP: Poly ADP-ribose polymerase
IDO: Indoleamine-2,3-dioxegenase
